# Knowledge into learning: comparing lecture, e‐learning and self‐study take‐home packet instructional methodologies with nurses

**DOI:** 10.1002/nop2.73

**Published:** 2016-12-20

**Authors:** Tracey Soper

**Affiliations:** ^1^ POETChamberlain College of NursingCelebrationFloridaUSA

**Keywords:** instructional, knowledge, nursing

## Abstract

**Aim:**

The aim of this quantitative experimental study was to examine which of three instructional methodologies of traditional lecture, online electronic learning (e‐learning) and self‐study take‐home packets are effective in knowledge acquisition of professional registered nurses.

**Design:**

A true experimental design was conducted to contrast the knowledge acquisition of 87 registered nurses randomly selected.

**Methods:**

A 40‐item Acute Coronary Syndrome (ACS) true/false test was used to measure knowledge acquisition. Based on 0.05 significance level, the ANOVA test revealed that there was no difference in knowledge acquisition by registered nurses based on which of three learning instructional method they were assigned. It can be concluded that while all of these instructional methods were equally effective in knowledge acquisition, these methods may not be equally cost‐ and time‐effective.

**Results:**

The study was able to determine that there were no significant differences in knowledge acquisition of nurses between the three instructional methodologies. The study also found that all groups scored at the acceptable level for certification. It can be concluded that all of these instructional methods were equally effective in knowledge acquisition but are not equally cost‐ and time‐effective. Therefore, hospital educators may wish to formulate policies regarding choice of instructional method that take into account the efficient use of nurses’ time and institutional resources.

## 1 Introduction

Instructional methodologies are educational approaches use for information sharing and learning (U.S. Naval Academy, 2010). Instructional methodologies provide the guidance for information delivery. This research involved comparing three instructional methodologies, lecture, teacher‐guided electronic (e‐learning) and self‐study take‐home packets. The intent of the research was to identify which of these three instructional methodologies produced the greatest knowledge acquisition in registered nurses required to complete a short‐term educational module about coronary artery disease. Nurses are a highly educated audience requiring an effective teaching‐learning delivery. Registered nurses employed in the acute care setting such as in hospital are required by the Joint Commission to be exposed to and demonstrate competency involving coronary artery disease.

While instructional methodologies provide the guidance for information delivery, imparting knowledge to students involves using different instructional methods to achieve the best possible results. Students may respond differently to various types of instructional methodologies that might influence greatly their knowledge acquisition (Roytek, 2010). Specifically, students differ in various ways, including the ways in which they retain information (Roytek, 2010). The research aimed to distinguish which of the three instructional methodologies of lecture, teacher‐guided electronic learning and self‐study take‐home packets had the most effective influence on learner's knowledge acquisition in a short‐term professional learning experience. Although many studies have sought to explore instructional teaching methods to optimize knowledge acquisition for registered nurses, none have concluded which is the best technique. The goal of this quantitative experimental research was to determine if there was a difference in knowledge acquisition of registered nurses enrolled in a short‐term professional development learning experience using three different instructional methodologies. This was an experimental study that contrasted identical course content delivered by three different instructional methodologies given to three randomly selected and randomly assigned groups of nurses. The study took place in a hospital in the South‐eastern USA. The results were expected to reveal the optimal learning method for human development officers to consider when planning similar short‐term professional development courses.

This research was relevant to modern society, which still uses the traditional lecture method of instructing students. The research might become an agent of change to learning institutions involved in the professional development of nurses. Policy makers interested in the efficacy of lecture, teacher‐guided e‐learning and self‐study take‐home packets as instruction methodologies may find this research helpful in formulating policies regarding the best instruction method for learning institutions. The significance of this research study was in addressing knowledge acquisition of nursing education in the 21st century that requires nurses to function with higher order thinking capabilities.

### 1.1 Literature review

#### 1.1.1 Lecture as the instruction method

The traditional education system has used the traditional instruction lecture method to enable students to acquire knowledge. According to Mikol (2005), nursing school instructors continue to lecture to the students despite the National League of Nursing Accreditation Council (NLNAC) promotion and innovation of new teaching strategies. The traditional lecture method involves the presence of students and teachers in a classroom; the teacher imparts knowledge by providing verbalization of the information to the student (Sterman et al., 2013). Through such a method, the student enjoys the teacher‐student interaction, which enhances learning (Sterman et al., 2013). The traditional lecture method of instructing students has ceased to be the sole manner of equipping students with necessary knowledge, although the method is not always cost‐ and time‐effective (Mickelson, Kaplan, & MacNeilly, 2009). The lecture method of instruction requires that nursing students and the instructor be together in a given venue in order for the learning process to occur (Mickelson et al., 2009).

Nurse educators have traditionally relied on a teacher‐centred lecture instructional model where the instructor is the content expert, while the nurse enrolees are the passive learners (Disch, 2012). This instructional model has been considered expensive, which many hospitals cannot financially afford (Mickelson et al., 2009). Due to limited financial resources, the nurses have the mandate to do multiple tasks for their patients, which hinders them from participating in a lecture classroom for continuous learning (Mickelson et al., 2009). Exorbitant health care costs have challenged the human development officer to find alternative teaching‐learning methodologies that are appropriate in a hospital setting (Roytek, 2010). The development of student‐centred learning in the academic setting may lend scientific placement in the hospital setting. Student‐centred learning focuses on the needs, abilities, interests and, learning styles of the students; the teacher acts only as a facilitator of learning (Disch, 2012). A student‐centred learning environment is primarily focused on the active role of the student; this environment makes the student responsible for their own learning (Disch, 2012).

#### 1.1.2 Electronic learning

New methods of instruction have emerged, with many learning institutions embracing advanced technology and modern instruction models (Abdelaziz, Kamel, Karam, & Abdelrahman, 2011). Student‐centred instruction provides learners with a flexible methodology of instruction, because the teacher and institution play a supportive role in which the student is in the foreground of the learning process (Disch, 2012). Adult learners have higher knowledge acquisition and retention when exposed to active student‐centred learning participation methodologies (Disch, 2012). As such, the lecturer is no longer the expert in an active learning environment.

With the acceptance of student‐centred instructional models and the technology revolution, e‐learning has emerged as a new form of delivering instruction. Many institutions have begun offering blended or hybrid models of instruction, which is a mixture of classroom and online instruction, or have moved towards exclusively online electronic learning (e‐learning; Dianati & Adib‐Hajbaghery, 2012). One form of e‐learning or virtual learning is teacher‐guided e‐learning. In this form of e‐learning, teachers provide personalized attention to learners when providing instruction. This instructional model allows instructors to interact with students online in addition to providing students with prepared materials. This combination of classroom techniques ensures that the students follow the correct path through the prepared learning materials (Gagnon, Gagnon, Desmartis, & Njoya, 2013).

At times, e‐learning also offers individualized instruction which print media cannot provide (Waldner, McGorry, & Widener, 2012). The lower cost and the presence supporting activities may be attractive to the human development officer. The advent of the Internet has increased the availability of coursework available 24/7 from any computer. Institutions of higher education and corporate training have been quick to adopt online learning. The mechanisms of distance learning traditionally involve packets that are emailed between the learning centre and the learner. The blending of the traditional homework assignments and the take‐home self‐paced adult learning packets was developed into the distance electronic learning environment supported by the Internet (Waldner et al., 2012). Another form of electronic learning may be found in Electronic Learning Aids (ELA), devices which promote hands‐on interaction with math, vocabulary and other lessons.

E‐learning is popular because of its potential for providing required learning contents online anytime, anywhere (Waldner et al., 2012). Frequently, the focus entails increasing the availability of learning experiences for learners who cannot or choose not to attend traditional face‐to‐face offerings. Moreover, from the perspective of instructional designers, assembling and disseminating electronic instructional content is more cost‐effective. E‐learning also enables instructors to handle more students while maintaining a learning outcome quality that is comparably equivalent to that of face‐to‐face instruction (Waldner et al., 2012).

#### 1.1.3 Self‐study take‐home packets as the instructional method

Adults display many learning styles in various situations (Cha & Kim, 2014). Self‐paced self‐study take‐home packets provide a form of instructional methodology that gives learners an opportunity to work individually according to their special needs. The advantage of take‐home packets is attractive to the self‐motivated learner who has already self‐identified knowledge gaps with a planned approach of gaining the missing knowledge (Cha & Kim, 2014). With self‐study, learners study by themselves using textbooks and hand out notes or lectures prepared by the teachers. However, self‐study limits the interactive face‐to‐face nature of the learning environment; this limits teachers’ guidance in critical thinking exercises, and thereby alters the nature of the teacher‐learner relationships. The lack of face‐to‐face interaction may be overcome by the package content delivery. Describing the learning or the necessary information in a story‐like manner simulates human interaction; thus, scenarios are an option when creating the humanistic paper learning approach. For the self‐motivated adult learner with clearly delineated learning goals and objectives, a self‐learning packet can support knowledge acquisition (Cha & Kim, 2014). According to Cha and Kim, nursing students are able to learn effectively with self‐study techniques.

### 1.2 Research cotext and questions

#### 1.2.1 Instruction method

The use of educational methods and classrooms teaching has spread across a variety of disciplines. Teachers use these methods for reaching their set goals and objectives. Educational methods, sometimes called techniques, are ways and means adopted by teacher to direct the learners’ activities towards an objective. Findings have shown that students have different classroom experiences because they approach learning and technology differently. This study cited several types of teaching methods: lecture, teacher‐guided electronic learning and self‐study take‐home packets.

An instructor implementing these components in a lecture is likely to produce student satisfaction. However, learners with a learning preference that includes visual, auditory or tactile may prefer electronic learning (Fleming, 1995). Electronic Learning Aids encourage hand‐on interaction and often focus on the learners’ special needs, which more often than not eliminate the tendency of boredom and promote a fun way of learning.

E‐learning is beneficial to learners in a way that they are able to share their learning perspectives online without the need to meet face‐to‐face. Unlike classroom lectures, online learning methods offer message sharing through chats, online discussion forms and public areas where students can post their information. Thus, online learning provides learning opportunities that are different from the traditional lecture method or take‐away packages.

Blended learning is the combination of online delivery of educational content with the best features of classroom interaction. This method includes live instruction to personalize learning, allow for thoughtful reflection and differentiate instruction from student to student across a diverse group of learners (Lloyd‐Smith, 2010). This method is found in the traditional classroom environment as well as online learning. The development of blended learning has led to changes in the education system, and the role of the teacher has changed along the way (Lloyd‐Smith, 2010).

#### 1.2.2 Research question and hypotheses

This study had a single research question which is as follows:

RQ1: Which teaching methodology—lecture, teacher‐guided electronic learning, or take‐home packets—results in the most knowledge acquisition by nurses in a short‐term professional developmental class required for the organizational continued certification?

The corresponding hypotheses were:

H(o): There was no difference in knowledge acquisition by registered nurses based on which of three learning instructional method they were assigned (lecture, teacher‐guided e‐learning or self‐study take‐home packet).

H(a): There was a difference in knowledge acquisition by registered nurses based on which of three learning instructional method they were assigned (lecture, teacher‐guided e‐learning or self‐study take‐home packet).

## 2 Methods

### 2.1 Research design

A two‐group experimental design is a research design used extensively in an experimental research considering behaviour and applied behaviour analysis of human or non‐human participants. All potential participants will complete the survey instrument developed for this proposed study. All completed surveys were used as data for this proposed research. In the case of this study, the focus is on the knowledge acquisition of nursing students after using audio recording in their online class. The first group considered in this study were those who undergone the learning instructional method of lecture. The second group considered in this study were those who undergone the learning instructional method of teacher‐guided e‐learning. The third group considered in this study were those who undergone the learning instructional method of self‐study take‐home packet.

Quantitative approaches are employed when the focus of the study is to determine relationships or differences between two or more variables (Babbie, 2012). Quantitative approaches make use of objective measures through numerical representations of the constructs considered in the study. For the purpose of this study, the dependent variables were the knowledge acquisition scores of the participants. The independent variable was the instruction method.

As opposed to a quantitative approach, the qualitative approach is focused on establishing “the meaning of a phenomenon from the views of participants” (Cozby, 2009, p. 20). A qualitative approach was considered for this research, but was found to be misaligned with the objective of this study because qualitative studies are not focused on comparing numerical scores of participants given an independent variable (Marshall & Rossman, 2011). Thus, a mathematical result is necessary in this research to properly test the research question. To achieve that mathematical result, a quantitative research design was considered for this study.

Two strategies for quantitative research were considered for this study. These two strategies involve the experimental and non‐experimental designs (Bryman, 2012). Experimental research involves controlling the environment through manipulating the independent variable and identifying both a treatment and a control group (Bryman, 2012). On the other hand, a non‐experimental research involves an environment that is not controlled by the researcher and the variables are measured as they occur in practice (Bryman, 2012). Cozby (2009) asserted that non‐experimental studies are appropriate when study participants are not subject to manipulation. However, for the purpose of this study, the participants were subject to different interventions. Thus, this is considered as an experimental study.

### 2.2 Data source

The sample population was comprised of 87 registered nurses in a Southeastern U.S. hospital specializing in acute care and chest pain accreditation, which totalled 200 registered nurses. G*Power was used to calculate the total sample size of at least 44, based on a medium effect size with 80% power. The sample (*n *=* *87) was randomly assigned into three cohorts, each consisted of 29 registered nurses.

Participants were selected using a systematic selection process, whereby every other individual was selected from a list of approximately 200 registered nurse caregivers at the hospital. The selected participants were randomly assigned to one of the three teaching modalities, by way of assigning every other person to groups A, B and C respectively. Each of the instructional methods were developed using identical goals and objectives, conveying the same information, involving approximately four hours of study and testing students with the same instrument. Twenty‐nine Registered Nurses completed their respective courses and reported on the acquired knowledge by taking the test.

The independent variable of the study was the instruction method. The three methods of instruction that were compared were lecture, teacher‐guided e‐learning and self‐study take‐home packets. The three types of teaching methods have similar goals and objectives, and each required four hours to learn the material. The same group of registered nursing curriculum experts developed all three courses. The dependent variable was knowledge acquisition, measured by the ACS quiz, which was a test developed for the course comprised of 40 true/false questions. The Acute Coronary Syndromes (ACS) quiz is an existing quiz questionnaire that will look at acute coronary syndromes and will test the respondents’ knowledge of the pathophysiology of these syndromes, the risk factors of these syndromes, the clinical presentations of these syndromes and the treatment and management of these syndromes. It is developed by the ACLS certification institution. The scores are obtained by counting the number of correct answers in the 40‐item questionnaire.

As the first part of the programme, before the groups started the classes, the participants signed the consent form before the data collection process commenced. This consent form addressed confidentiality issues that the registered nurses may have had regarding the research project. Signing the form served as an agreement to participate in the programme, given the assurance that no individual information would be disclosed to anyone else other than the researcher and the director of education. The informed consent form (Appendix S2) included the purpose of this research study, the expected participation, as well as the contact information of the researcher. The researcher was available to answer any questions the potential participants had regarding the study conduction. The researcher also emphasized that refusal to take part in the study would not have any negative consequences to the participant.

Moreover, if the participant felt the need to withdraw at any point during the course of data collection and analyses, he/she could have done so without any associated penalties. In the case of withdrawal, the participants’ initial responses were not considered in this study and they were treated as prospective participants who refused to participate in the study. Participants were also informed that there were no associated risks for participating in the study. Their responses used for research purposes were handled cautiously to ensure that these are kept confidential at all times. After all relevant information had been disclosed to the potential participants, they were presented with a consent form. If they chose to participate in the study, they were informed that they needed to return a signed copy of the consent form. If a signed copy of the consent form was not returned to the researcher, it was be assumed that the person did not wish to participate and he/she was no longer considered for the study.

All prospective participants who agreed to participate in the study were considered as part of the sample. Following the coursework, these nurses took a 40‐item test (Appendix S1) designed to evaluate knowledge acquisition using a true or false response scale. Each question was obtained directly from the course material. The test was distributed at the completion of each course under the supervision of a nurse educator. The exam for the lecture course was proctored by a hospital human development officer and administered immediately following the lecture. Each registered nurse was given one hour to complete the exam. The exam for participants using teacher‐guided electronic learning was taken as part of their last electronic lesson. These registered nurses were also allowed one hour to complete the test. The registered nurses who studied the take‐home packet took the exam under proctored settings the day after the packet was dispensed, and were allowed one hour to complete the test. The exam for each instruction methods was corrected by hand by the researcher using a template. An additional collection tool was used to collect demographic of job position of the registered nurses to display descriptive statistics contrasting the demographics of the three groups.

### 2.3 Data analysis

To analyse the data gathered, descriptive statistics and inferential statistics were conducted. Descriptive statistics were conducted to describe the samples and study the variables obtained. Frequencies and percentages were used to describe categorical data such as demographic characteristic of job position and the independent variable of method of instruction while measures of central tendencies such as mean and standard deviation were used to describe continuous variables such as the dependent variable of knowledge acquisitions. Normality testing of the study variables was also conducted using Kolmogorov‐Smirnov test.

To answer the research question in this study, Analysis of Variance (ANOVA) was conducted. ANOVA was conducted to determine if there is significance difference in the knowledge acquisition in the use of different instruction method. ANOVA was used since the independent variables are categorically measured variables that have more than two identified groups and there is only a single dependent variable (Babbie, 2012). Roberts, Wallace and Frances (2003) described analysis of variance (ANOVA) as a test that is used to compare means from three or more groups, where the critical values are obtained from the F‐distribution with the appropriate degrees of freedom. The independent variable of the study was the instruction method which has three methods of instruction of lecture, teacher‐guided e‐learning and self‐study take‐home packets. The dependent variable was knowledge acquisition, which was measured by the ACS, a test instrument developed for the course comprised of 40 true/false questions. A significance level of 0.05 was used in the analysis. There is significant difference in the knowledge acquisition if the *p*‐value is less than or equal to the level of significance value. In instances wherein the ANOVA determined significant relationships between independent and dependent variables, a post hoc Tukey's test of multiple comparison was also conducted to further identify the relationships between independent and dependent variables.

## 3 Results

### 3.1 Descriptive analysis

The target population for this research study was registered nurses in a Southeastern U.S. hospital specializing in acute care and chest pain accreditation. For this study, a total of 87 participants were sampled with professions ranging from registered nurses to nurse managers. Each of the three cohorts had 29 participants. The frequency and percentages of participants’ job positions are presented in Table 1. It can be observed that majority of the participants were registered nurses (*n *=* *80, 92%). There was one participant who was a nurse manager (1.1%) and one other participant who held a clinical position (1.1%).

**Table 1:** Frequency and percentages of participants by job position

Job position	Frequency	Percent
Registered Nurse	80	92.0
ARNP's	4	4.6
Nurse manager	1	1.1
Uncategorized	1	1.1
Other clinical	1	1.1
Total	87	100.0

Table 2 presents the frequency and percentages of participants to each of the three instructional methods. The instructional methodology considered in this research study consisted of three methods. These methods included lecture, teacher‐guided e‐learning and self‐study take‐home packets. An equal number of participants were randomly assigned to each of the three methods (*n *=* *29).

**Table 2:** Frequency and percentages of participants by instructional methods

Instructional method	Frequency	Percentage
E‐learning	29	33.3
Lecture	29	33.3
Self‐study	29	33.3
Total	87	100.0

Table 3 presents the descriptive statistics of the ACS quiz scores of participants. The 87 participants had a mean ACS score of 89.77 (*SD* = 6.51). It can be observed that the lowest score was 80, while the highest 100. This shows that participants in general had high scores in the ACS quiz.

**Table 3:** Descriptive statistics of ACS quiz score

	*N*	Minimum	Maximum	Mean	*SD*
ACS quiz score	87	80.00	100.00	89.77	6.51

To explore the distribution of participants’ job positions according to the three instructional methodologies, cross‐tabulation of data was performed. The results of the cross‐tabulation are presented in Table 4. It can be observed that among those participants assigned in the teacher‐guided electronic learning method, 28 were registered nurses (96.6%), while only one was an ARNP (3.4%). For the lecture, 27 participants were registered nurses (93.1%) while two were ARNPs (6.9%). In terms of those assigned to the self‐study, 25 (86.2%) were registered nurses, while the remaining four (13.2%) held positions as an ARNP, nurse manager and other clinical staff.

**Table 4:** Cross‐tabulation of job position and instructional methodology

Job position	Instructional methodology						Total	
	Electronic		Lecture		Self‐study			
	*n*	%	*n*	%	*n*	%	*N*	%
Uncategorized	0	0	0	0	1	3.4	1	1.1
Registered	28	96.6	27	93.1	25	86.2	80	92.0
ARNP	1	3.4	2	6.9	1	3.4	4	4.6
Nurse manager	0	0	0	0	1	3.4	1	1.1
Other clinical	0	0	0	0	1	3.4	1	1.1
Total	29	100.0	29	100.0	29	100.0	87	100.0

### 3.2 Normality testing

In order to examine whether parametric tests were appropriate for this research study, it was necessary to test whether the data followed a normal distribution. Table 5 presents the Kolmogorov‐Smirnov test conducted for normality on the dependent variable of this study. An alpha value of 0.05 was used for this analysis. Table 5 shows the following results: teacher‐guided e‐learning method (*K‐S *=* *1.029, *p* = 0.240), lecture method (*K‐S *=* *0.786, *p* = 0.567) and self‐study method (*K‐S *=* *0.832, *p* = 0.493). None of the tests reached significance, indicating that the data for each group were normally distributed.

**Table 5:** Kolmogorov‐Smirnov test for normality of ACS quiz scores according to instructional methods

	Electronic	Lecture	Self‐Study
(*N *= 87)	29	29	29
Normal parameters			
Mean	89.1379	91.0000	89.1724
*SD*	6.41830	6.08863	7.04105
Most extreme differences			
Absolute	0.191	0.146	0.154
Positive	0.191	0.114	0.154
Negative	−0.123	−0.146	−0.136
Kolmogorov‐Smirnov *Z* test	1.029	0.786	0.832
Asymp. Sig. (2‐tailed)	0.240	0.567	0.493

Table 5 also reports the mean score for the teacher‐guided e‐learning method as 89.14 (*SD* 6.42), the mean score for the lecture method as 91.00 (*SD* 6.09) and the mean score for self‐study as 89.17 (*SD* 7.04). From this table, it is observed that the lecture method resulted in the highest mean score while the teacher‐guided e‐learning method resulted to the lowest mean score. It should be noted that the difference between the highest and lowest score was minimal (1.86).

### 3.3 Hypothesis testing

Since the data were found to follow a normal distribution, a parametric test was selected. For the purpose of comparing the three cohorts of instruction method, a one‐way ANOVA test was performed. As observed in Table 6, it was determined that there was no significant difference in the scores between the three instructional methods (*F*(*2,86*) = 0.77, *p *=* *0.47). Thus, there is insufficient evidence to reject the null hypothesis, which states that there is no difference in knowledge acquisition by registered nurses, regardless of which of three learning instructional method they are assigned (lecture, teacher‐guided e‐learning or self‐study take‐home packet).

**Table 6:** ANOVA test to compare ACS quiz scores according to instructional methods

	Sum of squares	*df*	Mean Square	*F*	Sig.
Between groups	65.82	2	32.91	0.77	0.47
Within groups	3579.59	84	42.61		
Total	3645.40	86			

## 4 Discussion

To improve the use of existing knowledge and to facilitate more effective acquisition of new knowledge, nursing educational organizations require research in curricular models and pedagogies that depart from traditional lecture‐style learning paradigms. New methods of instruction have emerged, with many learning institutions embracing advanced technology and modern instruction models (Abdelaziz et al., 2011). These paradigms have long been the cornerstone of nursing education programmes. Evidence‐based research has demonstrated that students of normal intelligence may easily learn when proper instructional techniques and methodologies are applied (Kousar, 2010). However, the research about knowledge acquisition from specific instructional methodologies is limited. No matter the methodology, the instructor is instrumental in the implementation, the student relationships and the outcome. Therefore, understanding the most effective methodology may provide some insight for nursing educators to implement successful knowledge acquisition for RNs (Disch, 2012).

The conclusions made in this study may only be applicable to the registered nurses in the selected hospital, in short‐term certification courses and when comparing these three instructional methods. Adults display many learning styles in various situations (Cha & Kim, 2014). This current study was able to determine that there was no difference in knowledge acquisition by registered nurses based on which of three learning instructional method they were assigned (lecture, teacher‐guided e‐learning or self‐study take‐home packet). The result of this current study was different from that of an existing study by Roytek (2010) whom argued that students may respond differently to various types of instructional methodologies that might influence greatly their knowledge acquisition. Since the impact of differences of learning instructional method on the knowledge acquisition by registered nurses were insignificant, future research should choose the learning instructional methods that has the most positive impact on the time and financial resource of the health institution. Thus, future studies should consider the impact of the learning instructional method on the financial resource.

It is advised that future researchers include nurses in other hospitals and other practice settings, contrast other instructional methods, analyse finding with learning gain computed by giving a pretest as well as a posttest to nurses, and/or contrast these instructional methods in longer term courses. In addition to these suggestions, it is also advised that technician and other health professionals should be included in future studies regarding the optimal instructional method for learning acquisition in short term certification courses. It is also recommended that future studies examine not only immediate knowledge gain but retention of knowledge over a more extended period of time. Future studies should also include age demographics and test for generational differences.

It is advisable for future researchers to explore additional instructional approaches like DVD tutorials and stand‐alone video presentations that are known to facilitate acquisition of knowledge in a more cost‐effective and shorter period of time. Including these approaches in future research would help to determine whether these low cost instructional methods are equally efficient teaching methods for knowledge acquisition in short‐term certification courses.

It is further recommended that future researchers consider conducting qualitative studies exploring the thoughts and feelings of registered nurses in terms of their satisfaction with each of the methods. Although each of the three instructional methodologies resulted in the same knowledge acquisition to the registered nurses, it is presently unknown whether nurses had positive feelings towards each of the instructional methods. This might have effects on learning in future courses, should the administration move to a more cost‐effective method than presently used, which may not be well‐received by nurses.

## 5 Conclusion

The purpose of this quantitative study was to determine which of the three instructional methodologies of lecture, teacher‐guided e‐learning and self‐study take‐home packets provided the greatest knowledge acquisition in a short high‐stakes course for nurses. The study was able to determine that there were no significant differences in knowledge acquisition of nurses between the three instructional methodologies. The study also found that all groups scored at the acceptable level for certification. It can be concluded that all of these instructional methods were equally effective in knowledge acquisition, but they are not equally cost‐ and time‐effective. Therefore, hospital educators may wish to formulate policies regarding a choice of instructional method that takes into account the efficient use of nurses’ time and institutional resources.
