# Supplementary material for: Knowledge into learning: comparing lecture, e‐learning and self‐study take‐home packet instructional methodologies with nurses
Source: Nurs Open. 2016 Dec 20;4(2):76–83. doi: 10.1002/nop2.73 (PMC5340166; doi:10.1002/nop2.73)
Supplement: Supplementary file 1 [file NOP2-4-76-s001.docx]

**Appendix A:** **40-item Acute Coronary Syndrome (ACS) true/false test**

**Acute Coronary Syndromes—Managing Chest Pain and Coronary Heart Disease**

Quiz KEY 2010

True or False

1. Acute Coronary Syndrome, also called ACS, is an umbrella term used for any condition brought on by sudden reduced blood flow to the heart.  True or False
2. The leading cause of death in America is cancer; second is acute coronary syndrome of both men and women. True or False
3. The most common cause of ACS is high blood pressure with neurological deficits such as slurred speech, unstable gait, and weakness of hand grip strength.  True or False
4. There are known risk factors that are changeable and some that are not changeable; having high blood pressure and elevated cholesterol does not put you at higher risk of having heart vessel blockage. True or False
5. Acute coronary syndrome and coronary artery disease is the same thing in meaning, title, and context so you can use the words interchangeably. True or False
6. A family history of chest pain, heart disease or stroke is a risk factor for ACS.  True or False
7. Research indicates no difference in treatment and outcome of ACS related to gender or race.  True of False
8. A coronary vessel occlusion causes blockages in the vessel feeding the heart muscle; if a vessel gets blocked it can cause heart dysrhythmias, angina or heart attack.  True or False
9. The large artery on the right side of the heart is called the “widow maker” or the right coronary artery. True or False
10. Thank goodness the body has the ability to build collateral circulation that provides another source of oxygen enriched blood to the heart muscle as the native vessels clog up over time. True or False
11. During the past decade, heart attack survival has improved due to the use of thrombolytics. True or False
12. Emergency medical staff needs to know the difference between ST-Elevation heart attack (STEMI ) and non-ST-elevated (NSTEMI ) disease as one is treatable and the other is a death curse where no medical treatment can cure or control it. True or False
13. A Q wave is a layman’s term for death where the dead person’s tongue is sticking out of the side of the month signally death has occurred. True or False
14. The three goals of ACS management are: 1) weight control, 2) return to work, and 3) increasing the activities of daily living. True or False
15. Plaques, made up of fatty deposits and building up in the arteries, causing the arteries to narrow and decrease blood flow, is a common cause of ACS.  True or False
16. The risk for Acute Coronary Syndrome is equal in men and women, and begins to be a factor at the age of 40.  True or False
17. Stable Angina is predictable and diminishes with rest and the use of nitroglycerine, and does not necessarily indicate damage to the heart muscle.  True of False
18. Unstable Angina, lasting longer than 30 minutes, will cause a rapid increase in cardiac enzymes. True or False
19. Co- morbidities frequently seen with Coronary Heart Disease are hypertension, diabetes and vascular disease.  True or False
20. Important factors to consider when assessing chest pain include the duration of the pain, what the patient was doing when the pain started, and the severity of the pain.  True of False
21. When a patient has an MI, cardiac enzymes will slowly increase the first 48 hours after the infarct.  True or False
22. If a patient has high blood pressure and an increase in chest pain upon palpation, it strongly indicates the patient has Acute Coronary Syndrome.  True or False
23. When treating ACS, the 3 goals are increase coronary perfusion, decrease myocardial oxygen demand and decrease clot formation.  True or False
24. When providing nursing care to a patient with ACS, it is important to organize care and activity to allow for frequent rest periods.  True or False
25. Indications of low cardiac output include low blood pressure, increasing pulse rate, low urine output and decreased oxygen saturation.  True or False
26. ACS pain is frequently described as burning, squeezing, crushing, with the location usually the substernal area and it may radiate to the arm, neck, jaw or shoulder. True or False
27. Nitroglycerine reduces chest pain by decreasing blood flow, which reduces the workload of the heart.  True or False
28. Heart rate, heart contractility and coronary artery patency all effect the oxygenation (supply and demand) of an individual.  True or False
29. Monitoring fluid balance is vital when a patient has CHF or heart failure.  In ACS or AMI, monitoring fluid balance is not a necessity.  True or False
30. When a patient is suspected of an acute MI, interventions should include a 12-lead EKG, morphine, oxygen, nitroglycerine and aspirin (MONA).  True or False
31. Once a patient has been diagnosed with Acute Myocardial Infarct, it is too late to use cholesterol lowering medications.  True or False
32. When a patient has been hospitalized due to ACS, it is not appropriate to discuss smoking cessation because it produces too much stress for the patient.  True or False
33. Core Measures from the American College of Cardiology include aspirin upon arrival and prescribed at discharge, use of ACE inhibitors, Beta Blockers on arrival and prescribed at discharge and cardiac catheterization within 120 minutes of hospital arrival for acute MI. True of False
34. Ace Inhibitors dilate blood vessels to increase cardiac output and reduce loss of water and sodium.  True of False
35. Beta Blockers improve the heart’s ability to relax and decrease the heart rate, and over time should improve the overall pumping ability of the heart.  True or False
36. Anti-platelet medications are frequently ordered for people who cannot take aspirin or still get blood clots when taking aspirin; therefore they should never be used in combination with aspirin.  True or False
37. Nurses are not responsible to monitor ACC Guidelines/Core Measures regarding medication, because they are not responsible to order patient medications.  True or False
38. Important factors to include in discharge instructions after ACS include low fat diet, written and verbal instructions regarding medications, and education regarding warning signs and symptoms of a cardiac emergency and the actions to take.  True or False
39. Important lifestyle changes to discuss prior to discharge include smoking cessation, a heart-healthy diet, exercising and control of blood pressure and cholesterol.  True or False
40. According to the American Heart Association, about every 29 seconds an American suffers a coronary event, and about every minute, someone dies from one.  True or False

**Appendix B:**

**Acute Coronary Syndromes—Managing Chest Pain and Coronary Heart Disease**

**Education Department** 
 

**1/2011**

**Contents**

[Preface 3](https://mail.google.com/a/email.phoenix.edu/?ui=2&ik=4a798b4569&view=att&th=12e73a8597ad9776&attid=0.1&disp=vah&zw#0.1__Toc283377065#0.1__Toc283377065)

[Definition 3](https://mail.google.com/a/email.phoenix.edu/?ui=2&ik=4a798b4569&view=att&th=12e73a8597ad9776&attid=0.1&disp=vah&zw#0.1__Toc283377066#0.1__Toc283377066)

[Acute Coronary Syndrome 3](https://mail.google.com/a/email.phoenix.edu/?ui=2&ik=4a798b4569&view=att&th=12e73a8597ad9776&attid=0.1&disp=vah&zw#0.1__Toc283377067#0.1__Toc283377067)

[Objectives 4](https://mail.google.com/a/email.phoenix.edu/?ui=2&ik=4a798b4569&view=att&th=12e73a8597ad9776&attid=0.1&disp=vah&zw#0.1__Toc283377068#0.1__Toc283377068)

[ACS in the United States 5](https://mail.google.com/a/email.phoenix.edu/?ui=2&ik=4a798b4569&view=att&th=12e73a8597ad9776&attid=0.1&disp=vah&zw#0.1__Toc283377069#0.1__Toc283377069)

[Key Points 5](https://mail.google.com/a/email.phoenix.edu/?ui=2&ik=4a798b4569&view=att&th=12e73a8597ad9776&attid=0.1&disp=vah&zw#0.1__Toc283377070#0.1__Toc283377070)

Acute Coronary Syndromes [5](https://mail.google.com/a/email.phoenix.edu/?ui=2&ik=4a798b4569&view=att&th=12e73a8597ad9776&attid=0.1&disp=vah&zw#0.1__Toc283377071#0.1__Toc283377071)

[Causes 6](https://mail.google.com/a/email.phoenix.edu/?ui=2&ik=4a798b4569&view=att&th=12e73a8597ad9776&attid=0.1&disp=vah&zw#0.1__Toc283377072#0.1__Toc283377072)

[Risk Factors 6](https://mail.google.com/a/email.phoenix.edu/?ui=2&ik=4a798b4569&view=att&th=12e73a8597ad9776&attid=0.1&disp=vah&zw#0.1__Toc283377073#0.1__Toc283377073)

[Differences 7](https://mail.google.com/a/email.phoenix.edu/?ui=2&ik=4a798b4569&view=att&th=12e73a8597ad9776&attid=0.1&disp=vah&zw#0.1__Toc283377074#0.1__Toc283377074)

[Gender 7](https://mail.google.com/a/email.phoenix.edu/?ui=2&ik=4a798b4569&view=att&th=12e73a8597ad9776&attid=0.1&disp=vah&zw#0.1__Toc283377075#0.1__Toc283377075)

[Age 7](https://mail.google.com/a/email.phoenix.edu/?ui=2&ik=4a798b4569&view=att&th=12e73a8597ad9776&attid=0.1&disp=vah&zw#0.1__Toc283377076#0.1__Toc283377076)

[Race 7](https://mail.google.com/a/email.phoenix.edu/?ui=2&ik=4a798b4569&view=att&th=12e73a8597ad9776&attid=0.1&disp=vah&zw#0.1__Toc283377077#0.1__Toc283377077)

[Where are the Coronary Arteries? 8](https://mail.google.com/a/email.phoenix.edu/?ui=2&ik=4a798b4569&view=att&th=12e73a8597ad9776&attid=0.1&disp=vah&zw#0.1__Toc283377078#0.1__Toc283377078)

[Right Coronary Artery (RCA) 8](https://mail.google.com/a/email.phoenix.edu/?ui=2&ik=4a798b4569&view=att&th=12e73a8597ad9776&attid=0.1&disp=vah&zw#0.1__Toc283377079#0.1__Toc283377079)

[Left Main Coronary Artery 8](https://mail.google.com/a/email.phoenix.edu/?ui=2&ik=4a798b4569&view=att&th=12e73a8597ad9776&attid=0.1&disp=vah&zw#0.1__Toc283377080#0.1__Toc283377080)

[Coronary Occlusion 9](https://mail.google.com/a/email.phoenix.edu/?ui=2&ik=4a798b4569&view=att&th=12e73a8597ad9776&attid=0.1&disp=vah&zw#0.1__Toc283377081#0.1__Toc283377081)

[“The Widow Maker” 9](https://mail.google.com/a/email.phoenix.edu/?ui=2&ik=4a798b4569&view=att&th=12e73a8597ad9776&attid=0.1&disp=vah&zw#0.1__Toc283377082#0.1__Toc283377082)

[Left Anterior Descending 10](https://mail.google.com/a/email.phoenix.edu/?ui=2&ik=4a798b4569&view=att&th=12e73a8597ad9776&attid=0.1&disp=vah&zw#0.1__Toc283377083#0.1__Toc283377083)

[Left Circumflex 10](https://mail.google.com/a/email.phoenix.edu/?ui=2&ik=4a798b4569&view=att&th=12e73a8597ad9776&attid=0.1&disp=vah&zw#0.1__Toc283377084#0.1__Toc283377084)

[Obtuse Marginal Branch 11](https://mail.google.com/a/email.phoenix.edu/?ui=2&ik=4a798b4569&view=att&th=12e73a8597ad9776&attid=0.1&disp=vah&zw#0.1__Toc283377085#0.1__Toc283377085)

[Right Coronary Artery 11](https://mail.google.com/a/email.phoenix.edu/?ui=2&ik=4a798b4569&view=att&th=12e73a8597ad9776&attid=0.1&disp=vah&zw#0.1__Toc283377086#0.1__Toc283377086)

[Collateral Circulation 12](https://mail.google.com/a/email.phoenix.edu/?ui=2&ik=4a798b4569&view=att&th=12e73a8597ad9776&attid=0.1&disp=vah&zw#0.1__Toc283377087#0.1__Toc283377087)

[Unstable Angina 12](https://mail.google.com/a/email.phoenix.edu/?ui=2&ik=4a798b4569&view=att&th=12e73a8597ad9776&attid=0.1&disp=vah&zw#0.1__Toc283377088#0.1__Toc283377088)

[Non-ST-Elevation MI (NSTEMI) 13](https://mail.google.com/a/email.phoenix.edu/?ui=2&ik=4a798b4569&view=att&th=12e73a8597ad9776&attid=0.1&disp=vah&zw#0.1__Toc283377089#0.1__Toc283377089)

[ST- Elevation MI (STEMI) 13](https://mail.google.com/a/email.phoenix.edu/?ui=2&ik=4a798b4569&view=att&th=12e73a8597ad9776&attid=0.1&disp=vah&zw#0.1__Toc283377090#0.1__Toc283377090)

[Stable Angina 14](https://mail.google.com/a/email.phoenix.edu/?ui=2&ik=4a798b4569&view=att&th=12e73a8597ad9776&attid=0.1&disp=vah&zw#0.1__Toc283377091#0.1__Toc283377091)

[Initial Assessment 14](https://mail.google.com/a/email.phoenix.edu/?ui=2&ik=4a798b4569&view=att&th=12e73a8597ad9776&attid=0.1&disp=vah&zw#0.1__Toc283377092#0.1__Toc283377092)

[History 14](https://mail.google.com/a/email.phoenix.edu/?ui=2&ik=4a798b4569&view=att&th=12e73a8597ad9776&attid=0.1&disp=vah&zw#0.1__Toc283377093#0.1__Toc283377093)

[Physical Exam 14](https://mail.google.com/a/email.phoenix.edu/?ui=2&ik=4a798b4569&view=att&th=12e73a8597ad9776&attid=0.1&disp=vah&zw#0.1__Toc283377094#0.1__Toc283377094)

[Breath Sounds 14](https://mail.google.com/a/email.phoenix.edu/?ui=2&ik=4a798b4569&view=att&th=12e73a8597ad9776&attid=0.1&disp=vah&zw#0.1__Toc283377095#0.1__Toc283377095)

[Lubb Dub 15](https://mail.google.com/a/email.phoenix.edu/?ui=2&ik=4a798b4569&view=att&th=12e73a8597ad9776&attid=0.1&disp=vah&zw#0.1__Toc283377096#0.1__Toc283377096)

[Pain Assessment 15](https://mail.google.com/a/email.phoenix.edu/?ui=2&ik=4a798b4569&view=att&th=12e73a8597ad9776&attid=0.1&disp=vah&zw#0.1__Toc283377097#0.1__Toc283377097)

P-Q-R-S-T method [15](https://mail.google.com/a/email.phoenix.edu/?ui=2&ik=4a798b4569&view=att&th=12e73a8597ad9776&attid=0.1&disp=vah&zw#0.1__Toc283377098#0.1__Toc283377098)

[ACS Pain Description 16](https://mail.google.com/a/email.phoenix.edu/?ui=2&ik=4a798b4569&view=att&th=12e73a8597ad9776&attid=0.1&disp=vah&zw#0.1__Toc283377099#0.1__Toc283377099)

Ask Yourself [16](https://mail.google.com/a/email.phoenix.edu/?ui=2&ik=4a798b4569&view=att&th=12e73a8597ad9776&attid=0.1&disp=vah&zw#0.1__Toc283377100#0.1__Toc283377100)

[Summary – EKG changes 17](https://mail.google.com/a/email.phoenix.edu/?ui=2&ik=4a798b4569&view=att&th=12e73a8597ad9776&attid=0.1&disp=vah&zw#0.1__Toc283377101#0.1__Toc283377101)

[Anterior Infarction 18](https://mail.google.com/a/email.phoenix.edu/?ui=2&ik=4a798b4569&view=att&th=12e73a8597ad9776&attid=0.1&disp=vah&zw#0.1__Toc283377102#0.1__Toc283377102)

[Lateral Infarction 18](https://mail.google.com/a/email.phoenix.edu/?ui=2&ik=4a798b4569&view=att&th=12e73a8597ad9776&attid=0.1&disp=vah&zw#0.1__Toc283377103#0.1__Toc283377103)

[Inferior Infarction 18](https://mail.google.com/a/email.phoenix.edu/?ui=2&ik=4a798b4569&view=att&th=12e73a8597ad9776&attid=0.1&disp=vah&zw#0.1__Toc283377104#0.1__Toc283377104)

[Right Ventricular Infarction 18](https://mail.google.com/a/email.phoenix.edu/?ui=2&ik=4a798b4569&view=att&th=12e73a8597ad9776&attid=0.1&disp=vah&zw#0.1__Toc283377105#0.1__Toc283377105)

[Posterior Infarction 18](https://mail.google.com/a/email.phoenix.edu/?ui=2&ik=4a798b4569&view=att&th=12e73a8597ad9776&attid=0.1&disp=vah&zw#0.1__Toc283377106#0.1__Toc283377106)

[Bundle Branch Block 19](https://mail.google.com/a/email.phoenix.edu/?ui=2&ik=4a798b4569&view=att&th=12e73a8597ad9776&attid=0.1&disp=vah&zw#0.1__Toc283377107#0.1__Toc283377107)

[Diagnostic Blood Tests 19](https://mail.google.com/a/email.phoenix.edu/?ui=2&ik=4a798b4569&view=att&th=12e73a8597ad9776&attid=0.1&disp=vah&zw#0.1__Toc283377108#0.1__Toc283377108)

[At Risk for ACS 20](https://mail.google.com/a/email.phoenix.edu/?ui=2&ik=4a798b4569&view=att&th=12e73a8597ad9776&attid=0.1&disp=vah&zw#0.1__Toc283377109#0.1__Toc283377109)

[3 Goals of ACS Management 20](https://mail.google.com/a/email.phoenix.edu/?ui=2&ik=4a798b4569&view=att&th=12e73a8597ad9776&attid=0.1&disp=vah&zw#0.1__Toc283377110#0.1__Toc283377110)

[Factors Influencing the Heart’s Oxygen Supply and Demand 21](https://mail.google.com/a/email.phoenix.edu/?ui=2&ik=4a798b4569&view=att&th=12e73a8597ad9776&attid=0.1&disp=vah&zw#0.1__Toc283377111#0.1__Toc283377111)

[The Nurse 22](https://mail.google.com/a/email.phoenix.edu/?ui=2&ik=4a798b4569&view=att&th=12e73a8597ad9776&attid=0.1&disp=vah&zw#0.1__Toc283377112#0.1__Toc283377112)

[Acute MI Interventions 23](https://mail.google.com/a/email.phoenix.edu/?ui=2&ik=4a798b4569&view=att&th=12e73a8597ad9776&attid=0.1&disp=vah&zw#0.1__Toc283377113#0.1__Toc283377113)

[Early Invasive Management Cardiac Catheterization 23](https://mail.google.com/a/email.phoenix.edu/?ui=2&ik=4a798b4569&view=att&th=12e73a8597ad9776&attid=0.1&disp=vah&zw#0.1__Toc283377114#0.1__Toc283377114)

[ACC/AHA 24](https://mail.google.com/a/email.phoenix.edu/?ui=2&ik=4a798b4569&view=att&th=12e73a8597ad9776&attid=0.1&disp=vah&zw#0.1__Toc283377115#0.1__Toc283377115)

[Core Measures from the ACC 24](https://mail.google.com/a/email.phoenix.edu/?ui=2&ik=4a798b4569&view=att&th=12e73a8597ad9776&attid=0.1&disp=vah&zw#0.1__Toc283377116#0.1__Toc283377116)

[ACE Inhibitors 24](https://mail.google.com/a/email.phoenix.edu/?ui=2&ik=4a798b4569&view=att&th=12e73a8597ad9776&attid=0.1&disp=vah&zw#0.1__Toc283377117#0.1__Toc283377117)

[Angiotensin II Receptor Blockers 24](https://mail.google.com/a/email.phoenix.edu/?ui=2&ik=4a798b4569&view=att&th=12e73a8597ad9776&attid=0.1&disp=vah&zw#0.1__Toc283377118#0.1__Toc283377118)

[Beta Blockers 25](https://mail.google.com/a/email.phoenix.edu/?ui=2&ik=4a798b4569&view=att&th=12e73a8597ad9776&attid=0.1&disp=vah&zw#0.1__Toc283377119#0.1__Toc283377119)

[Other Medications 25](https://mail.google.com/a/email.phoenix.edu/?ui=2&ik=4a798b4569&view=att&th=12e73a8597ad9776&attid=0.1&disp=vah&zw#0.1__Toc283377120#0.1__Toc283377120)

[Anti-Platelets 25](https://mail.google.com/a/email.phoenix.edu/?ui=2&ik=4a798b4569&view=att&th=12e73a8597ad9776&attid=0.1&disp=vah&zw#0.1__Toc283377121#0.1__Toc283377121)

[Not Recommended 25](https://mail.google.com/a/email.phoenix.edu/?ui=2&ik=4a798b4569&view=att&th=12e73a8597ad9776&attid=0.1&disp=vah&zw#0.1__Toc283377122#0.1__Toc283377122)

[Be Proactive 26](https://mail.google.com/a/email.phoenix.edu/?ui=2&ik=4a798b4569&view=att&th=12e73a8597ad9776&attid=0.1&disp=vah&zw#0.1__Toc283377123#0.1__Toc283377123)

[ACS Discharge Instructions 27](https://mail.google.com/a/email.phoenix.edu/?ui=2&ik=4a798b4569&view=att&th=12e73a8597ad9776&attid=0.1&disp=vah&zw#0.1__Toc283377124#0.1__Toc283377124)

[Lifestyle Changes 27](https://mail.google.com/a/email.phoenix.edu/?ui=2&ik=4a798b4569&view=att&th=12e73a8597ad9776&attid=0.1&disp=vah&zw#0.1__Toc283377125#0.1__Toc283377125)

[Reference 28](https://mail.google.com/a/email.phoenix.edu/?ui=2&ik=4a798b4569&view=att&th=12e73a8597ad9776&attid=0.1&disp=vah&zw#0.1__Toc283377126#0.1__Toc283377126)

Preface

The Education Department encompasses system-wide education and training responsibilities targeting large or specific associate groups and the organization as a whole. The education department has developed specific programs along with having a collaborative relationship with community, to provide orientation and training to nurses and technicians working in our chest pain center. Unit based specific critical competencies require validation prior to the associate giving performance of care. The Education Department offers to local EMS the opportunity to enroll and come to all cardiac educational classes.   

Definition

Acute Coronary Syndrome

Acute coronary syndrome is a term used for any condition brought on by sudden, reduced blood flow to the heart. Acute coronary syndrome can describe chest pain you feel during a heart attack or chest pain you feel while you're at rest or doing light physical activity (unstable angina). Acute coronary syndrome is usually diagnosed in an emergency room or hospital (Mayo Clinic, 2010).

Objectives

1. **Define Acute Coronary Syndrome (ACS).**
2. **Identify what the initial assessments and interventions for a person with ACS and acute myocardial infarction (AMI).**
3. **Differentiate the gender and age related differences in the symptoms of ACS.**
4. **Understand the causes and co-morbidities in the symptoms of ACS.**
5. **Review anatomy coronary arteries.**
6. **Review path physiology ACS.**
7. **Identify the various types and locations of AMI and their representation on 12 lead electrocardiograms.**
8. **Differentiate between left ventricular and right ventricular AMI.**
9. **Identify assessment and interventions on arrival to emergency department.**
10. **Identify and differentiate medications and treatment options for STEMI vs. NSTEMI.**

ACS in the United States

- Acute coronary syndrome (ACS) is the leading cause of morbidity and mortality among both men and women in the United States, affecting more than 13.9 million people.
- Acute myocardial infarction (AMI) is most dramatic of presentations.
- AMI affects approximately 1.1 million people in the United States. The mortality rate with AMI is approximately 30%.
- About once every 29 seconds, an American suffers a coronary event, and about every minute, someone dies from one.

**Key Points**

- All ACS have some degree of coronary artery occlusion
- The most common cause of an ACS is thrombus or plaque formation within a coronary artery
- The degree and duration of occlusion dictates the type of ACS

 
 
 
 

*(American Heart Association, 2010)*

**Causes**

Acute coronary syndrome may develop slowly over time by the building up of plaques in the arteries in your heart. These plaques, made up of fatty deposits, which cause the arteries to narrow and make it more difficult for blood to flow through them. This buildup of plaques is known as atherosclerosis. Eventually, this buildup means that your heart can't pump enough oxygen-rich blood to the rest of your body, causing chest pain (angina) or a heart attack.

Another medical term closely related to acute coronary syndrome is coronary artery disease. Coronary artery disease refers to the damage to your heart arteries from atherosclerosis.

If one of the plaques in your coronary arteries ruptures, it can cause a heart attack. In fact, many instances of coronary artery syndrome develop after a plaque ruptures. A blood clot will form on the site of the rupture, blocking the flow of blood through the artery (Mayo Clinic, 2010).

**Risk Factors**

The risk factors for acute coronary syndrome are similar to those for other types of heart disease. Acute coronary syndrome risk factors include:

- Older age (over 45 for men and over 55 for women)
- High blood pressure
- High blood cholesterol
- Cigarette smoking
- Lack of physical activity
- Type 2 diabetes
- Family history of chest pain, heart disease or stroke

(Mayo Clinic, 2010)

**Differences**

**Gender**

Heart disease doesn't discriminate. It is the leading killer of men and women. But when it comes to diagnosing and treating it, there is a gender gap. Women with heart attacks are more likely to die than men...and that's not just older women. Women of all ages are more likely to die. According to one Israeli study that adjusted for age, size and other factors, the death risk for women was 1.7 times that of men (Hsia, 2007).

Studies have shown that women with chest pain wait too long before heading to the emergency room. Thrombolytics work better in men than in women? Large Studies have found that women's survival improves with these drugs, but not to the same extent as men, though it is not known why. Coronary artery disease is the leading cause of death in women. More than twice as many women die from cardiovascular disease as from all forms of cancer combined. Evaluation for suspected coronary disease differs in women because of frequently misleading results provided by treadmill testing without imaging (Hsia, 2007).

**Age**

The incidence of acute coronary syndrome increases with age. Older patients with acute coronary syndrome are most likely to present with atypical symptoms. Many elderly patients with acute coronary syndrome do not receive evidence-based therapies. This situation emphasizes the importance of improving quality-of-care programs to reinforce the use of therapies among elderly individuals (American Heart Association, 2010).

**Race**

No racial predilection is observed. Racial disparities in treatment and outcome have been noted (American Heart Association, 2010).

**Where are the Coronary Arteries?**

The heart receives its own supply of blood from the coronary arteries. Two major coronary arteries branch off from the aorta near the point where the aorta and the left ventricle meet. These arteries and their branches supply all parts of the heart muscle with blood.

**Right Coronary Artery (RCA)**

The right coronary artery branches into:

- Right marginal artery
- Posterior descending artery

The right coronary artery supplies:

- right atrium
- right ventricle
- bottom portion of both ventricles and back of the septum

**Left Main Coronary Artery (also called the left main trunk)**

The left main coronary artery branches into:

- Circumflex artery
- Left Anterior Descending artery (LAD)

The left coronary arteries supply:

- Circumflex artery - supplies blood to the left atrium, side and back of the left ventricle
- Left Anterior Descending artery (LAD) - supplies the front and bottom of the left ventricle and the front of the septum

**(Cleveland Clinic, 2010)**

**Coronary Occlusion**

- Newly formed plaques tend to be more unstable and are more likely to break open (rupture).

- If a plaque ruptures, a blood clot may form and suddenly block the blood flow to your heart muscle

- This block causes chest pain and may cause an ACS.

- The degree and duration of occlusion determines the type of ACS
  - Degree – the larger the blockage the worse it is
  - Duration – a new occlusion or sudden increase in occlusion size is bad

**“The Widow Maker”**

**Left Anterior Descending**

 

**Left Circumflex**

 

**Obtuse Marginal Branch**

- Part of the Circumflex artery
- Supplies blood to the side and back of the left ventricle

(Lewis, 2007) 
 
 

**Right Coronary Artery**

 
 

**Collateral Circulation**

- Sometimes collateral circulation develops to provide another source of oxygen-rich blood to the deprived heart muscle.

- Collateral circulation is tiny branches off the coronary arteries that develop to "bypass" the area of narrowing and help to restore blood flow.

- Adequate unless the heart requires increased oxygen, such as during exercise or in stressful situation.

(Lewis, 2007)

**Unstable Angina**

**Non-ST-Elevation MI (NSTEMI)**

- Partial occlusion of a coronary artery

- Chest pain lasting more than 20 minutes

- No ST-elevation on EKG

- Diagnosed using elevated cardiac enzymes

American Heart Association, 2010)

**ST- Elevation MI (STEMI)**

 

 

**Stable Angina (not an ACS)**

- Is predictable and diminishes after taking nitroglycerin and resting.

- Although stable angina can be disturbing, it does not necessarily indicate heart muscle damage and can occur for years without harm.

**Initial Assessment**

**History**

- **Most important** – prior history of CAD, MI
- Co-morbidities such as hypertension, diabetes, or vascular diseases
- Age
- Sex

**Physical Exam**

- General appearance
- Vital signs
- Breath sounds
- Heart auscultation
- Pain assessment

**Breath Sounds**

- Rule-out other causes of chest pain
- Auscultate for equal breath sounds bilaterally
  - Non-cardiac causes of chest discomfort include pneumothorax, pulmonary embolism
- New or worsening crackles
  - Pulmonary edema
  - Worsening CHF

(Lewis, 2007)

**Lubb Dub: Best Spots for Auscultation!!**

- **Atrial** (S2) – “dub”; 2^nd^ intercostal space at the right sternal border
- **Pulmonic** – 2^nd^/3^rd^ intercostal space at the left sternal border
- **Tricuspid** - 4^th^/5^th^ intercostal space at the left sternal border
- **Mitral** (S1) – “lubb”; apical pulse; 5^th^ intercostal space on the midclavicular line

**Pain Assessment**

(Lewis, 2007)

**ACS Pain Description**

(American Heart Association, 2010)

**Summary – EKG changes**

MendlM

| **Anterior Infarction** | |
| --- | --- |
|  | - ST elevation without abnormal Q wave - Usually associated with occlusion of the left anterior descending branch of the left coronary artery (LCA) |

| **Lateral Infarction** | |
| --- | --- |
|  | |
|  | - ST elevation with/without abnormal Q wave - May be a component of a multiple-site infarction - Usually associated with obstruction of the left circumflex artery |

| **Inferior Infarction** | |
| --- | --- |
|  | - ST elevation with/without abnormal Q wave - Usually associated with right coronary artery (RCA) occlusion |

| **Right Ventricular Infarction** | |
| --- | --- |
|  | - Usually accompanies inferior MI due to proximal occlusion of the RCA - Best diagnosed by 1 - 2 mm ST elevation in lead V4R - An important cause of hypotension in inferior MI recognized by jugular venous distension with clear lung fields - Aggressive therapy is indicated, including: reperfusion, adequate IV fluids for right heart filling, and pacing to maintain A-V synchrony if necessary |

| **Posterior Infarction** | |
| --- | --- |
|  | - Tall, broad (>0.04 sec) R wave and ST depression in V1 and V2 (reciprocal changes) - Frequently associated with inferior MI - Usually associated with obstruction of RCA and or left circumflex coronary artery |

(Mendler, 1997)

**Bundle Branch Block**

The last 0.04 seconds of deflection on the QRS complex is used to determine the direction of the block. In V1 or MCL1, if the QRS duration is greater than 0.12 seconds (usually 0.14 - 0.20 seconds) and the last 0.04 second segment of the complex is pointing down (negative deflection), the block is LEFT. If the last 0.04 seconds of the QRS complex is pointing up and is positively deflected, the block is RIGHT

- In LBBB, the ST segment is difficult or impossible to evaluate

- Take into account the patient’s previous EKG (if available), the patient’s symptoms, and blood work

- The finding or development of a ***new LBBB*** warrants a diagnosis of ST-elevation

(Mendler, 1997)

**Diagnostic Blood Tests**

- Serum laboratory tests drawn at specific intervals
  - Determining actual time of ischemia or injury
  - Monitoring healing process

- Consider:
  - Time to rise
  - Peak
  - Return to normal

**At Risk for ACS**

**3 Goals of ACS Management**

- **Increase coronary perfusion**
  - Revascularize the coronary artery
  - Preserve myocardial tissue

- **Decrease myocardial oxygen demand**
  - Reduce cardiac workload

- **Decrease clot formation**

(Mayo Clinic, 2010)

**Factors Influencing the Heart’s Oxygen Supply and Demand**

**The Nurse**

- Careful observation for any ischemic changes on the EKG

- Frequent status checks for indications of low cardiac output
  - Decreased blood pressure
  - Increased heart rate
  - Low urine output
  - Decreased oxygen saturation
  - Arrhythmias

- Organize care and activities to allow rest period

- Gradually increase activity level and assess tolerance

- Know the ACS medications –  monitor response to these medications

- Daily weights and I&O = fluid balance.  Rule out CHF role in chest discomfort

- Teach and reinforce teaching every day

- Reacts quickly to changes in patient status:  EKG, vital signs, chest discomfort

- Gives a good report –  to co-workers and to MD

- A thorough report - includes ejection fraction, fluid balance, ACS medications, risk factors for Coronary Heart Disease, and plan for care

**Acute MI Interventions**

- 12-lead EKG

- Cardiac enzymes

- ACLS teaches us MONA:
  - Morphine
  - Oxygen
  - Nitroglycerin
  - Aspirin

- Beta Blockers, Ace Inhibitors

- Cardiac catheterization, possible surgery

**Early Invasive Management Cardiac Catheterization**

**ACC/AHA**

- Absolute standard of cardiology care are guidelines from the **American College of Cardiology/American Heart Association**  derived from Evidence Based Medicine

**Core Measures from the ACC**

- Aspirin on arrival and prescribed at discharge
- ACE Inhibitor or ARB for LVSD
- Smoking Cessation Advice/Counseling
- Beta Blocker on arrival and prescribed at discharge
- PCI Received Within 120 Minutes of Hospital Arrival (for AMI)
- ACE Inhibitor or ARB
- Beta Blocker
- Aspirin
- Other useful medications
- Nitrates
- Anti-platelets other than aspirin
- Cholesterol lowering medications

**ACE Inhibitors**

- ‘Inhibit’ the conversion of angiotensin I to angiotensin II, a potent vasoconstrictor
- Decrease aldosterone secretion and, therefore, sodium and water retention
- Dilate your blood vessels to improve the amount of blood your heart pumps (cardiac output) and to lower blood pressure.
- Increase blood flow, which helps to decrease the amount of work your heart has to do.

**Angiotensin II Receptor Blockers**

- Alternative to ACE Inhibitor
- Same effects as ACE inhibitors but their action mechanism is slightly different
- Use as an ACE alternative if patient develops or has contraindications to ACE:
  - Persistent cough
  - Acute renal failure

**Beta Blockers**

- Improve the heart's ability to relax
- Slow the heart rate
- Reduce angina
- Over time, beta-blockers improve the heart's overall pumping ability

**Other Medications**

- Nitrates
  - Vasodilate veins and arteries to reduce heart’s workload
  - Relieve chest pain and other symptoms of angina.
- Calcium channel blockers
  - Slow heart rate and lower blood pressure to reduce heart’s workload
  - Dilate coronary arteries and reduce angina
- Cholesterol-lowering
  - Lower blood cholesterol and may reduce risk of a future heart attack.

**Anti-Platelets**

- Used for people who cannot take aspirin or who still get blood clots while on aspirin therapy.
- May be used in combination with aspirin
- These medications are commonly given for at least one month and in some cases longer, after stents have been placed in one or more coronary arteries.

**Not Recommended**

- Hormone replacement therapy (HRT) in women increases complications and death in women at risk for cardiovascular disease
- Doctors may prescribe short-term HRT to manage menopause symptoms.  Calculating a woman’s individual risk factors weigh into this decision.

**Be Proactive**

 

**NOT a Contraindication**

A contraindication to taking an ACE Inhibitor, Beta Blocker, or Aspirin is ***not***….

“Because the doctor didn’t order it”

Nurses either know or find out why the med is not ordered.  Check this out as soon as you notice the patient isn’t on a recommended med – don’t wait until discharge.

**Round With Doctor**

- Don’t wait for the doctor to ask….**Just do it!**
- Ask questions
- Find out what the plan is

**ACS Discharge Instructions**

- Counseling about a low sodium (2 gram) and/or low fat diet
- Medications – verbal and written information
- Education on cholesterol level and ejection fraction (the patient’s actual numbers)
- Smoking cessation information
- We need to provide the patient with written information prior to discharge that discuss the following:
- Diet
- Activity
- Medication
- Hazards of smoking
- High blood pressure
- Warning signs and symptoms of a heart attack and action to take if they occur

**Lifestyle Changes**

- No matter what medical treatment a patient receives, lifestyle changes are critical to managing the disease.
  - Smoking cessation
  - Heart healthy diet
  - Exercising
  - Control of blood pressure and cholesterol

**Reference**

American Heart Association. (2010). Acute Coronary Syndrome. Retrieved from

<http://www.americanheart.org/presenter.jhtml?identifier=3010002>.

Cleveland Clinic. (2010). Your coronary arteries? Retrieved from

<http://my.clevelandclinic.org/heart/disorders/cad/cad_arteries.aspx>

Hsia, J. (2007). Gender differences. Retrieved from

<http://www.womensheart.org/content/HeartDisease/gender_differences.asp>.

Lewis, S., Heitkemper, M., Dirksen, S., O’Brien, P., Bucher, L. (2007). Medical

     surgical nursing: Assessment and management of clinical problems. (7^th^ ed.).

     Mosby, St. Louis. Section 7. p. 739-842.

Mayo Clinic. (2010). Acute coronary syndrome. Retrieved from

<http://www.mayoclinic.com>.

Mendler, M. (1997). 12 Lead E.C.G. Field diagnoses made easy. Retrieved from

[*http://www.publicsafety.net/12lead_dx.htm*](http://www.publicsafety.net/12lead_dx.htm).
